# Cerebro Spinal Meningitis

**Published:** 1866-05

**Authors:** E. S. Gaillard

**Affiliations:** Richmond, Virginia


ART. IV.—*Cerebro Spinal Meningitis*—By E. S. GAILLARD, M. D., Richmond, Virginia.

No one would so do violence to the accuracy and safety of Medical literature, as to attempt an exhaustive essay upon a subject proverbially obscure, and in regard to which so little is satisfactorily or definitely known.

There are few diseases which the physician is called upon to treat, in relation to which even the best medical libraries furnish such limited information, and the largest professional circles such confused, contradictory and perplexing testimony.

The object of the writer, therefore, is not to discuss the subject in extenso, but leaving this fruitful labor to more competent hands, curiously to examine the facts that, during the last four years, have been brought prominently to his attention.

The history of this disease is limited in extent, and, if governed by its teachings, we are to conclude that either this obscure affection is of modern origin, or that early writers failed to appreciate its existence.

Dr. G. A. Moses, who has made careful examinations of all accessible authorities upon this subject, states that "the first recorded appearance of cerebro-spinal meningitis occurred in France, in 1310. It did not attract attention again until 1503; a disease almost similar appeared in 1516 aud 1517. After a very severe winter (1553) in Silesia, it carried off large numbers of the population. In 1580, associated as now with catarrhal affections, it killed no less than 10,000 in Rome, 12,000 in Madrid and proportionately large numbers in other cities.

"During the civil wars in France, Ozenaur says 'the armies, Catholic and Protestant, are decimated by a new disease, the subjects being attacked with a sudden and furious pain in the head.' It lasted more than three months and but few were saved."

"Sydenham reports it in 1661, as selecting the young and most robust subjects, and partaking of the character of Typhus."

In 1778, during an epidemic of typhus in Lyle, a disease appeared in all respects similar.

The disease, in more recent years, has appeared at various points throughout the United States, and, during the war, severe epidemics visited many points of interest in the Southern States. At Bowling Green, Grenada, Mississippi, New Orleans, Mobile, Charleston, around Richmond, and in different parts of Virginia, the disease prevailed with much malignity.

It was not until 1830 that any name was given to this disease, and though autoposies do not invariably reveal lesions of the brain and cord, such injuries are, however, sufficiently uniform to warant the use of the name selected.

Of the pathology of the disease, but little can be said. Speculations and unsatisfactory arguments have been repeatedly offered, but it is not expedient or useful to present them.

Testimony and recorded facts only can be accepted as reliable premises in the argument, and, until these are placed in possession of the profession, it is unprofitable to expend attention, either speculative or deductive, upon this subdivision of the subject.

The diagnosis of the disease is certainly difficult, unless there be an epidemic prevailing and suspicion on this account be relatively aroused.

The most experienced and vigilant practitioner is frequently deceived, and so no one need blush to acknowledge that a case of cerebro-spinal meningitis has terminated fatally in his hands before any suspicion of the true nature of the malady had been aroused. So many have been mortified and censured, in this connection, that it is well to place this important fact prominently on record.

The symptoms of the disease vary in accordance with the character of the attack. In some cases, where there are no true lesions of the nerve centres (with consequent loss or impairment of sensation and motion) but with inflammation of the meninges, the symptoms are neither as alarming nor as pronounced, as is universally observed in the grave invasions of the disease.

There is just reason for believing, that inflammation of the nerve centres frequently and fatally occurs without actual lesion, death, it is presumed, being due, in such cases, to one of the many complications that are apt to supervene. This fact will explain the great diversity of testimony in the necrology of this disease. Able and competent examiners have failed to find, not unfrequently, evidences of actual lesion in the nerve centres, and have consequently denied not only the existence of these lesions, but the pathological deductions that have been based upon them.

The prominent and interesting fact, in this connection, is, that these lesions are observed in a large proportion of the autopsies of this disease, while death frequently occurs, from supervening causes which produce no such results.

Whether in fatal or non-fatal attacks of this disease, the symptoms are usually, if not invariably, declared suddenly—sometimes after a meal which has been evidently and fully enjoyed. In differential diagnosis, this fact is entitled to due weight.

There is usually prodromic headache, occurring for a brief period, followed sometimes in an hour, by a chill or distinct rigor, with vomiting and purging. This last symptom is occasionally so marked and malignant in character as to entail a rapid prostration, with collapse and many of the symptoms of cholera. The patient usually, but not uniformly, soon becomes stupid, and his intellect is manifestly impaired. This condition passes sometimes into complete stupor. The headache increases, and acute pain is felt about the base of the brain. Convulsions occasionally occur, but usually the stiffness of the muscles of the neck and back, which is early apparent, rapidly increases, and, without marked convulsion, the patient passes into a condition of opisthotonos.

The pupils are sluggish in action, and trismus occasionally occurs. The reaction is seldom marked, and heat of skin, though sometimes apparent, is an exceptional phenomenon. Pains in the joints and abdomen are frequently a cause of acute suffering. The tongue, at first natural in most cases, is soon covered with fur, and then assumes, in addition to the manifestations observed in typhoid complications, a swollen and distorted appearance. In the Southern States, there has very seldom been manifested the diarrhœa, comparative or colliquative, observed in Europe. On the contrary, constipation has very frequently been obstinate and persistent. Dysphagia and difficulty of speech are frequently marked, many physicians regarding the last symptom as pathognomic.

The pulse is usually, after the first day, small and easily compressible; at first, natural or nearly so; next, it is increased in frequency; this condition subsiding, it frequently becomes abnormally slow, and continuing to be soft and compressible, it fully assumes the thready character that is the herald of dissolution. The ratio, normally existing between the frequency of the pulse and respiration, is generally subverted, and the respiration soon becomes slow and labored.

The skin is usually relaxed, and not unfrequently there is marked diaphoresis. Thirst is incessant. It becomes more and more difficult to arouse the patient, and finally hopeless coma supervenes, with the prodromic phenomena of approaching death.

It will be readily appreciated that, in a disease manifesting such a diversity of symptoms, with but little uniformity usually apparent, one fails to readily and promptly diagnosticate the true pathological condition of the patient.

Nothing of a satisfactory character is known as to the etiology of the disease, the cause assigned being, not unfrequently, as diverse and unconnected as are the localities of prevalence. Depressing agencies, hereditary, dietetic hygienic and climatic seem to be all predisposing and immediate causes of the disease. It prevailed at the South during the past four years, usually in winter and among those subjects immediately to its rigors, in connection with crude and insufficient diet. As a rule, in the camps, even where the attendant circumstances were similar in regard to diet and exposure, the negroes were the first to suffer, and exhibited the largest mortality. This may be due to that natural want of resiliency which, as is familiar to those who have attended them in sickness, is a physiological peculiarity of the race.

This disease prevails sporadically; though, as a rule, it makes its appearance in the form of an epidemic, and, like all epidemics, it not unfrequently wears a varied livery.

No satisfactory evidence of its contagiousness has ever been presented; though, from its epidemic prevalence, many have been alarmed and induced to fly from imaginary contagion. It is well that the fact of its never having proved to be in the least contagious should be impressed upon the minds of all, when the disease makes its appearance.

The period of incubation is certainly unknown. The evidence so far on this subject is valueless.

Whether as seen in the States of the Gulf, or as in Virginia and Tennessee, in cities or villages, on the seaside or mountain side, in malarial or mountain air, in the pure atmosphere of the country or amid the depressing agencies of cities, the course of the disease seems unaffected by surrounding circumstances. The white soldier on the Rapidan or the negro laborer in Mobile, manifested usually the same symptoms, and the mountain air, surrounding the one, seemed as little to influence the result, as did the seaside atmosphere which enveloped the other. It may be said, then, that city or country atmospheres, cæteris paribus, do not alter the results.

There is one remarkable feature of this disase, as it has prevailed in the Southern States, and that is (contrary to its recorded history elsewhere), as many adults as children have been the subjects of its attack.

European writers dwell with some emphasis upon the fact, that, as a rule, children under twelve years of age were almost exclusively affected, and of these children all, or very nearly all, were males.

It is remarkable that the invasion of this disease is confined almost exclusively to the male sex. In the many cases visited, examined or reported, there was not a single instance in which the female, either as an adult or child, had been attacked.

There are no complications so frequently presented as to be regarded as special in character; though, in many cases, complicacations of hereditary, hygienic or latitudinal origin are observed.

There seems to be no disposition to a recurrence of the disease during convalescence, and generally the patient advances slowly, tediously, but regularly, to the normal condition existing previous to its invasion.

There are no sequelæ observed, deserving of special mention.

The prognosis in this disease is uniformly grave and disheartening. Indeed, one may reasonably expect to see most cases die, and to be surprised, as well as gratified, at each recovery.

In France, through a series of years, the mortality was often eighty-five per cent., and frequently far beyond this. A few practitioners have met with better success, but their records are but little less disheartening. Some of these death-statistics are as follows: 66 of 154; 24 of 40; 122 of 195, etc. Dr. S. C. Young states, that in the epidemic at Grenada, Mississippi, of thirty-five cases, there was not a single recovery.

Though no statistics of the epidemic in the Gulf States have escaped destruction, it may safely be said that the mortality in this disease, throughout these States, has not been less than sixty to eighty per cent. Dr. Moses, of Mobile, who saw very many cases, states that he did not witness a single recovery, and heard of but five. In the majority of fatal cases, death takes place before the fifth day, though occasionally the patient lives until the tenth, and, in rare instances, until the twelfth day.

Few diseases are so frequently fatal, and, as a rule, one would seldom err if he expected death.

M. Gaussaud states that he lost but two cases in one hundred and sixty-two, and ascribes this good result to the fact that, regarding the disease as a cephalagic fever, due to miasmatic origin, he treated all of his cases with quinine. It is extremely improbable that the disease seen in this country can be due to such a cause, or that, with such treatment, the recorded result would be in any respect altered. Most of the epidemics seen in the Southern States occurred in winter, when the air was pure and certainly uncontaminated by any malarial taint. There may have been sporadic cases in summer, and possibly an epidemic at this season, but, if so, the facts have never been mentioned by any of the numerous medical gentlemen interested in the study and investigation of the subject. That the disease prevailed in summer, in the localities mentioned, is certainly very improbable, and no one, so far seen, for a moment entertained the idea that its etiology bears the least relation to malarial or miasmatic poison; nor did the use of quinine produce any material results.

It must be borne in mind, that the mortality mentioned in those cases, where the disease is clearly pronounced.

There are cases in which there is meningeal inflammation, without loss of motion, sensation or speech, and, in such cases, the number of recoveries is very much greater. Where there is opisthotonos, or trismus, or loss of speech or motion, it may reasonably be expected that not more than eighty in one hundred cases will recover.

In treatment, the physician must expect to be disheartened and disappointed. It would be time uselessly spent to record the varied forms of treatment which have been faithfully and fruitlessly instituted. In this disease no one has any special remedy to recommend.

Routinists, Empirics and Professional Egotists meet here, on common ground, with the scientific practitioners of the country, confessing a common disappointment and mortifying inefficiency. Clinical experience in this disease accomplishes at least one good result, in being the grave of presumption and therapeutic fanaticism.

It is generally conceded, that the most advisable form of treatment is the free use of the lancet and the scarificator, *before* any evidences of prostration or collapse are manifested.

The physician seldom sees a patient in time to institute such treatment, but when the opportunity occurs, experience certainly demands a prompt and fearless resort to it.

The use of mercury, both in full doses at the commencement of the disease, and afterwards for its constitutional effect, is admitted to be the most reliable form of treatment that can be adopted. The general testimony, both in Europe and in America, warrants the early and persistent use of this remedy.

Stimulants should be used, but there is no doubt that in the treatment of this disease, as in that of many others, the prevailing tendency is to use the remedies unnecessarily and injuriously. Where stimulation is required, the use of rubefacients, with hot applications to the cutaneous surface, will most beneficially protect the brain and nervous system from the influences of alcohol, given, very commonly, with poisonous and overwhelming frequency!

In this disease, the centre and citadel of animal life are assailed, and it is certainly culpable to recklessly subject them to any influences that lessen the powers of successful resistance. Alcohol is certainly the therapeutic boon, but most physicians have seen its injudicious use destroy the last manifestations of nervous resiliency.

While, therefore, stimulation is frequently, if not universally, required, it should be most cautiously and attentively instituted, and the adjuvants of friction and hot applications rigidly enjoined.

Opium may sometimes be admissible to relieve suffering and procure rest, but it should be carefully and guardedly used.

Chloroform, given to a partial extent by inhalation, will be found to relieve the suffering occasioned by muscular spasm, but, for manifest reasons, this should be administered only when the exigency of the case really demands it.

In this disease, as in most others, many incidental symptoms will be manifested requiring attention, but these will, of course, be treated on general principles.

In the autopsies, where death has occurred, with manifest lesion of the nervous centres, the phenomena apparent are so uniform in character, that, after a single inspection of the brain and its meninges, at the table, or after seeing a correct drawing of them, the scalpel will alone often solve the mystery of an unintelligible death.

The post-mortem appearances of the brain, in death from this disease, are characteristic, and it is therefore with special satisfaction and pleasure, that a correct representation of them is presented in the chromo-lithograph furnished with this article.

This drawing was made at the table, and to Dr. G. A. Moses due acknowledgement is made for the opportunity of presenting it to the members of the profession. So far as is known, there is no drawing of this character accessible, (if one has ever been presented) and it is believed that the plate presented, both in delineation and coloring, is so accurate, that any one familiar with it would be enabled to identify the disease, whose ravages are there depicted.

The dura mater is usually uninjured, while the arachnoid, both cerebral and spinal, almost invariably furnishes evidences of acute inflammation.

The large veins of the pia mater are turgid and much congested, and seen through the coat of lymph and greenish yellow pus, present an arborescent appearance, marked and distinct. This greenish yellow pus and lymph are also seen on the surface of the encephalon, and at the base of the brain it is found in quantity. It is also found in the subarachnoidal space of the cord, and it envelops the cord very generally.

The substance of the brain and cord is, so far as is known, seldom injured, though at times there is some effusion in the ventricles, and incipient evidences of softening in the nervous substances; as a rule, however, the substance of the brain and cord is found uninjured.

It is to be regretted, that, even after the sad and disheartening experience of so many epidemics, our knowledge in regard to this disease is so limited and unsatisfactory. Its etiology and pathology are equally obscure, and its livery so varied, as to render identification difficult and sometimes impossible. Its treatment is most perplexing and disheartening. The sick man lies doomed and helpless before us, and after comparatively few hours of just alarm and conscious agony, passes into the dream-land of blessed delirium. Disease mercifully administers that "sweet oblivous antidote" which, in his conscious moments, he vainly craved from man, and soon even the ignorant bystander recognizes the fact that—

> "The life of all this blood
> Is touched corruptively, and his poor brain
> Doth, by the idle comments that it makes
> Foretell the ending of mortality."

